# Xerostomia and hyposalivation among a 65‐yr‐old population living in Oslo, Norway

**DOI:** 10.1111/eos.12757

**Published:** 2021-01-27

**Authors:** My Tien Diep, Janicke Liaaen Jensen, Rasa Skudutyte‐Rysstad, Alix Young, Anne Thea Tveit Sødal, Beáta Éva Petrovski, Lene Hystad Hove

**Affiliations:** ^1^ Department of Cariology and Gerodontology Faculty of Dentistry University of Oslo Oslo Norway; ^2^ Department of Oral Surgery and Oral Medicine Faculty of Dentistry University of Oslo Oslo Norway; ^3^ Faculty of Dentistry University of Oslo Oslo Norway

**Keywords:** epidemiology, mouth dryness, saliva

## Abstract

This study aimed to describe the prevalence and associated factors for xerostomia and hyposalivation in a young‐elderly population. A random sample of 460 65‐yr‐old people living in Oslo, Norway, answered a questionnaire and underwent a clinical examination (237 men and 223 women; response rate 58%). Ten percent of respondents reported xerostomia. The median Summated Xerostomia Index was 6 (interquartile range [IQR]: 5–7) and the median Clinical Oral Dryness Score was 2 (IQR: 1–3). The median unstimulated whole saliva (UWS) secretion rate was 0.34 (IQR: 0.20–0.53) mL min^–1^ and the median stimulated whole saliva (SWS) secretion rate was 1.74 (IQR: 1.24–2.38) mL min^–1^. In 8% of the study participants the UWS secretion rate was ≤0.1 mL min^–1^ and in 4% the SWS secretion rate was ≤0.7 mL min^–1^. Three percent of the study participants had both xerostomia and hyposalivation with respect to UWS. Xerostomia was significantly associated with medication use, having rheumatic disease, and having received radiation therapy to the head/neck region. Hyposalivation with respect to UWS and SWS was significantly associated with medication use and type II diabetes. Even though xerostomia and hyposalivation were not prevalent conditions in this population, clinicians should be especially aware of the salivary conditions in patients taking four or more medications, patients diagnosed with type II diabetes, and those who have undergone radiation therapy to the head/neck region.

## INTRODUCTION

In many societies, the proportion of elderly people is gradually increasing (1). Many elderly people have declining general and oral health, and an increasing proportion of older people with general and oral health challenges may lead to higher individual and societal costs (2). It is therefore important to map the oral health status of the ‘young elderly’ in order to plan for future dental health needs and services as they age.

Saliva is important in maintaining a healthy oral cavity because it lubricates the oral surfaces, rinses the mouth, and neutralizes acids, and thus protects against caries and erosive wear, as well as mucosal infections. Reduced salivary secretion can cause problems with eating, speaking, and wearing dental prostheses (3, 4, 5). Having an adequate volume of saliva is therefore crucial for good oral health‐related quality of life.

Salivary secretion rate is commonly determined by collecting unstimulated and/or stimulated whole saliva using a standardized protocol. If the secretion rates measured are below designated thresholds, the patient is diagnosed with ‘hyposalivation’ (6). Epidemiological studies show a varying prevalence of hyposalivation among the elderly, potentially because of different definitions and measurement methods (6, 7). In addition, social demographics and medical background characteristics may influence salivary conditions. Furthermore, it has been reported that women, in general, have lower salivary secretion rates than men (8), and hyposalivation is associated with female gender (9, 10, 11), use of xerogenic medications (9, 12), increasing age (13), and chronic diseases, such as diabetes and Sjögren's syndrome (14).

Hyposalivation may lead to xerostomia, the sensation of dry mouth. However, hyposalivation and xerostomia are not necessarily correlated (15). Information on xerostomia is obtained by interviews or questionnaires. A variety of questions with predetermined response alternatives have been used for this purpose: some only determine the presence of dry mouth (‘Does your mouth feel dry?’) (9), whereas others explore the extent of dry mouth (‘How often does your mouth feel dry?’) (10), as well as investigate the problems related to dry mouth (‘Do you have difficulty with swallowing?’) (5). A comprehensive approach is to use the Xerostomia Inventory, a multi‐item questionnaire developed to measure the severity of chronic xerostomia (16). The original questionnaire was shortened to the Summated Xerostomia Inventory‐Dutch Version (17).

Previous studies have shown that the prevalence of xerostomia increases with age (12, 13, 18, 19). Xerostomia is reported more commonly in women (10, 18, 19, 20), smokers (12, 20), and individuals with symptoms of depression (9, 20). Xerostomia is also associated with impaired general health (12, 21) and use of medication (12) – in particular, a high number of medications (19) or xerogenic medications (20). Individuals with xerostomia also tend to report reduced oral health‐related quality of life (21, 22, 23).

In most developed countries, 65 yr of age is the accepted beginning of old age, although there is no general agreement (24). Accordingly, 65 yr of age can be considered as the threshold age for the group ‘young elderly’.

Even though saliva is important for maintaining good oral health, few studies focus on both subjective and objective salivary conditions among the young‐elderly population. The aims of this study were therefore to determine the prevalence of xerostomia and hyposalivation among 65‐yr‐old people living in Oslo, Norway, to explore the correlation between the two conditions, and to investigate their association with gender, smoking, education, medical conditions, and medication use in this population.

## MATERIAL AND METHODS

### Study design and setting

The data presented in this cross‐sectional study were part of a larger study investigating oral health in 65‐yr‐old people in Oslo, Norway (the OM65‐study). The participants were examined at the Research Clinic of the Institute of Clinical Dentistry, University of Oslo, between 26 February 2019 and 13 December 2019. The study protocol was approved by the Norwegian Regional Committee for Research Ethics (REK 2018/1383) and was performed in compliance with the tenets of the Declaration of Helsinki. Prior to study inclusion, all participants signed a written informed consent form, including a plain language statement.

### Participants

The target population was 65‐yr‐old (born in 1954) residents of Oslo, Norway. Eligible individuals were randomly selected from the Norwegian tax registry, and invitation letters were sent to 1230 individuals. No later than 2 wk after sending the invitation letters, the individuals were contacted by telephone and asked if they were interested in participating in the study.

### Questionnaire

The self‐administered questionnaire was sent to the participants via an electronic link to an online questionnaire program (Nettskjema; University of Oslo). Participants answered the questionnaire prior to attending the clinical examination. Xerostomia was assessed using the standardized question ‘How often does your mouth feel dry?’, with response categories ‘Never’, ‘Occasionally’, ‘Frequently’, and ‘Always’ (10, 25). Those who reported dry mouth ‘Frequently’ or ‘Always’ were grouped as ‘xerostomic’.

Subjective dry mouth symptoms were assessed further using the Summated Xerostomia Inventory‐Dutch Version (SXI‐D), which contains five questions related to dry mouth. The SXI‐D sum score ranges between 5 and 15, with a higher score representing an increased number of symptoms and/or an increased frequency of symptoms related to dry mouth (17).

The participants were also asked about smoking habits, their highest level of completed education, whether they had previously received radiation therapy in the head/neck region, how many medications they use regularly, and whether they had type II diabetes or rheumatic disease (subgroups not specified). Use of medications was categorized into ‘no medications’, ‘1–3 medications’, and ‘≥4 medications’. The participants’ level of education was dichotomized into ‘higher education’ (university/college education) and ‘basic education’ (high school, elementary school, or lower).

### Clinical examinations

Participants were instructed to refrain from eating, drinking, and smoking for at least 1 h before the clinical examination. Standardized sialometry was performed on all participants between 8 am and 3 pm. For collection of unstimulated whole saliva (UWS), participants were instructed to sit relaxed and swallow any saliva in their mouth. During the 5 min saliva‐collection period, the participants were asked to avoid swallowing saliva by spitting regularly into a test cup. After 5 min, each participant was asked to spit any remaining saliva into the test cup. For collection of stimulated whole saliva (SWS), the participants were first instructed to chew on a paraffin wax tablet (Ivoclar Vivadent) for 30 s, and then to swallow the saliva that was produced. Participants were then instructed to continue to chew on the wax tablet for a further period of 5 min and to spit out all saliva, produced regularly, into a fresh test cup. The test cups were preweighed and chilled on ice, and saliva samples were weighed after sample collection. The assumed density of saliva was 1 g mL^–1^. In this study, hyposalivation was defined as a salivary secretion rate of ≤0.1 mL min^–1^ for UWS (20, 26) and of ≤0.7 mL/min^–1^ for SWS (20).

Objective oral dryness was also assessed clinically using the Clinical Oral Dryness Score (CODS) (27, 28). In this scoring system, 10 signs of oral dryness are evaluated (score range 0–10; higher scores represent more severe dryness), including mirror tests and visual signs of mucosal wetness, presence and frothiness of saliva, and presence of cervical caries and debris. This examination was performed after UWS sampling and before SWS sampling.

### Statistical analyses

Data were collected in the Oral Data Collector sheet specifically designed for data entry in this study, developed in Microsoft Excel 2016 (Microsoft) and imported into stata (Stata version 16.1; StataCorp) for statistical analyses. Descriptive statistical analyses were performed and the results are presented in the form of number (*n*) with percentage or median with interquartile range (IQR). All data were stored, and analyses performed, in the TSD (Service for Sensitive Data, Centre for Information Technology Services, University of Oslo).

Chi‐square and Fisher's exact tests were used to determine any differences in the distribution of categorical variables. As the continuous variables did not follow a normal distribution, Kruskal–Wallis ANOVA and the Mann–Whitney *U*‐test were used to detect differences in median values of continuous, numerical variables between two or three groups. Spearman rank correlation analysis was used to measure the strength and direction of the linear relationships between the parameters used to determine dry mouth.

As the number of medications taken showed a significant association with both xerostomia and hyposalivation, this variable was chosen as the main factor for further investigation using regression analysis. Gender, education level, smoking habits, presence of type II diabetes or rheumatic disease, and experience with radiation therapy to the head/neck were all explored as confounding factors. However, only factors that were significantly associated with the outcome variable in the multivariate analysis were included in the final model. To study the relationship between xerostomia and the number of medications taken, univariate and multivariate logistic regression were used, and data are presented in the form of unadjusted and adjusted ORs with their 95% CI. To study the relationship between UWS and SWS secretion rates and the number of medications taken, univariate and multivariate linear regression were used. As a result of the high number of unusual and influential data (outliers), failure of the data to follow a normal distribution, and heteroscedasticity of the residuals, square root transformation was applied, and linear regression with robust function was used. The data are presented in the form of crude and adjusted β‐coefficients, with their 95% CI. The level of significance was set to *P* < 0.05.

## RESULTS

Of the 797 eligible participants who both received a letter and were contacted by telephone, 460 attended the examination (response rate 58%). Three of the attendees did not answer the questionnaire and were therefore excluded from the analyses. The sociodemographic and medical background characteristics of the study population (*n* = 457) are presented in Table 1.

**TABLE 1:** Sociodemographic and medical background characteristics of the study population.

Characteristic	*n* (%)
All	457 (100)
Gender	
Male	236 (52)
Female	221 (48)
Education level	
Higher education	305 (67)
Basic education	152 (33)
Smoking	
Current	50 (11)
Former	210 (46)
Never	197 (43)
Medications (no.)	
≥4	117 (26)
1–3	216 (47)
0	124 (27)
Type II diabetes	
Yes	31 (7)
No	426 (93)
Rheumatic disease	
Yes	56 (12)
No	401 (88)
Radiation head/neck^a^	
Yes	7 (2)
No	450 (98)

^a^ Experience with radiation therapy to the head and neck area.

### Subjective dry mouth parameters

Data on the prevalence of subjective dry mouth and related factors are presented in Table 2. Ninety percent of the participants reported having dry mouth ‘never’ or ‘occasionally’. The presence of symptoms of dry mouth according to the standard xerostomia question was significantly associated with the number of medications taken, rheumatic disease, and radiation therapy to the head/neck region. A feeling of dry mouth ‘frequently’ or ‘always’ was significantly more common among those who used ≥4 medications (17%), than those who used no or 1–3 medications (4% and 9%, respectively).

**TABLE 2:** Subjective dry mouth parameters according to gender, education level, smoking, and general health factors.

Characteristic	Xerostomia (frequently/always)	SXI‐D score
All	45 (10)	6 (5–7)
Gender		
Male	21 (9)	**6 (5**–**7)**
Female	24 (11)	7 (6–8)
Education level		
Higher education	25 (8)	**6 (5**–**7)**
Basic education	20 (13)	7 (6–8)
Smoking		
Current	4 (8)	7 (6–8)
Former	23 (11)	6 (5–8)
Never	18 (9)	6 (5–7)
Medications (no.)		
≥4	**20 (17)**	**7 (6**–**8)**
1–3	20 (9)	6 (5–7)
0	5 (4)	6 (5–7)
Type II diabetes		
Yes	3 (10)	7 (5–8)
No	42 (10)	6 (5–7)
Rheumatic disease		
Yes	**10 (18)**	**7 (6**–**8)**
No	35 (9)	6 (5–7)
Radiation head/neck		
Yes	**4 (57)**	**11 (7**–**15)**
No	41 (9)	6 (5–7)

Values are given as *n* (%) or median (interquartile range). Total number of study participants = 457.

Values shown in bold text are statistically significant (*P* < 0.05: chi‐square/Fisher's exact, Kruskal–Wallis, or Mann–Whitney *U* test, as appropriate).

Abbreviations: SXI‐D, Summated Xerostomia Inventory‐Dutch Version.

The median SXI‐D score was 6, and 95% of the participants had a score of less than 11. Although there were significant differences in many of the comparisons in relation to the median SXI‐D score, the most pronounced difference was between individuals who had undergone radiation therapy to the head/neck region and those who had not (median SXI‐D score: 11 vs. 6).

### Objective dry mouth parameters

**FIGURE 1:**
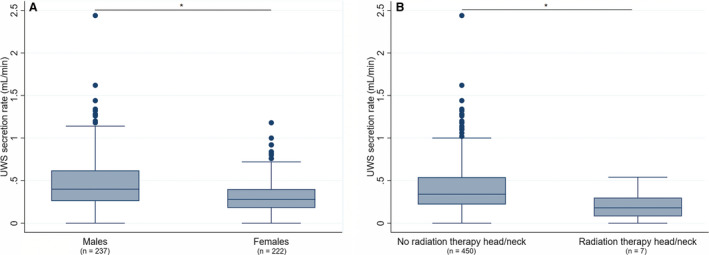
Unstimulated whole saliva (UWS) secretion rate according to gender (A) and radiation therapy to the head/neck area (B). Boxplots illustrate the distribution of UWS secretion rate, with each box showing median, interquartile range, and upper and lower quartiles. Dots in the figure represent outliers. *P < 0.05, Mann–Whitney *U*‐test.

The overall median UWS secretion rate was 0.34 (0.20–0.53) mL min^–1^. It was significantly higher in male participants (0.40 mL min^–1^) than in female participants (0.28 mL min^–1^) and significantly lower in individuals who had undergone radiation therapy to the head/neck region (0.18 mL min^–1^) than in those who had not (0.34 mL min^–1^) (Figure 1). Overall, 8% of the participants had hyposalivation with respect to UWS (≤0.1 mL min^–1^), and the condition was significantly associated with the number of medications used and type II diabetes (Table 3). Hyposalivation with respect to UWS was significantly more common among those who took ≥4 medications (13%) than in those who took no medications (5%).

**TABLE 3:** Objective dry mouth parameters according to gender, education level, smoking, and general health factors.

Characteristic	Hyposalivation UWS	Hyposalivation SWS^a^	CODS
All	36 (8)	18 (4)	2 (1–3)
Gender			
Male	17 (7)	**15 (6)**	2 (1–3)
Female	19 (9)	3 (1)	2 (1–3)
Education			
Higher education	24 (8)	13 (4)	2 (1–3)
Basic education	12 (8)	5 (3)	2 (1–3)
Smoking			
Current	6 (12)	**5 (10)**	**2 (2**–**3)**
Former	14 (7)	4 (2)	2 (1–3)
Never	16 (8)	9 (5)	2 (1–3)
Medications (no.)			
≥4	**15 (13)**	**9 (8)**	2 (1–3)
1–3	15 (7)	5 (2)	2 (1–3)
0	6 (5)	4 (3)	2 (1–3)
Type II diabetes			
Yes	**6 (19)**	**5 (16)**	2 (1–4)
No	30 (7)	13 (3)	2 (1–3)
Rheumatic disease			
Yes	6 (11)	1 (2)	2 (1–3)
No	30 (7)	17 (4)	2 (1–3)
Radiation head/neck			
Yes	2 (29)	1 (14)	**3 (2**–**6)**
No	34 (8)	17 (4)	2 (1–3)

Values are given as *n* (%) or median (interquartile range). Total number of study participants = 457.

Values shown in bold text are statistically significant (*P* < 0.05: chi‐square/Fisher's exact, Kruskal–Wallis, or Mann–Whitney *U* test, as appropriate.

Abbreviations: CODS, Clinical Oral Dryness Score; SWS, stimulated whole saliva secretion rate ≤0.7 mL min^–1^; UWS, unstimulated whole saliva secretion rate ≤0.1 mL min^–1^.

^a^ Date were missing for eight study participants.

**FIGURE 2:**
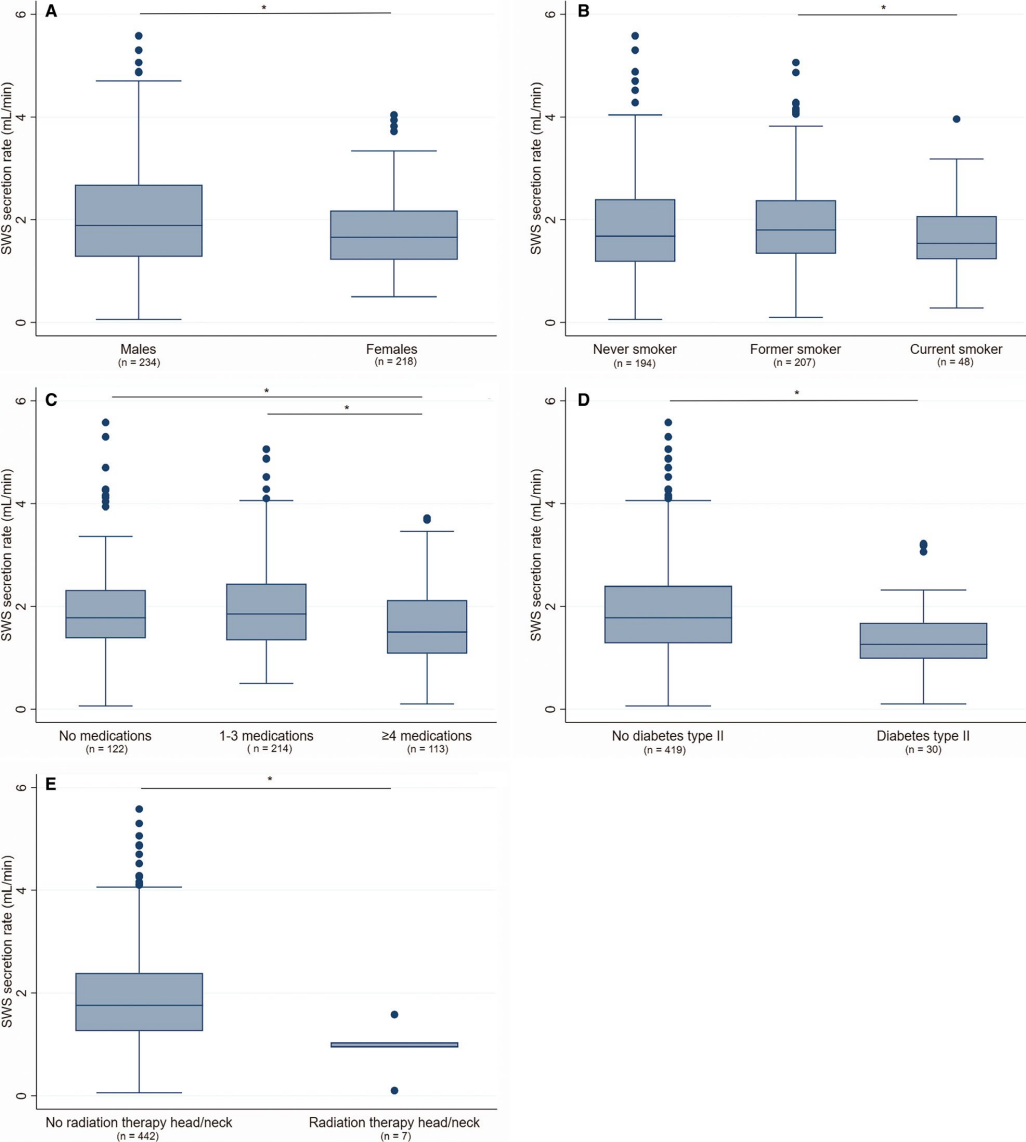
Stimulated whole saliva (SWS) secretion rate according to gender (A), smoking habits (B), medications (C), diabetes (D), and radiation therapy to the head/neck area (E). Boxplots illustrate the distribution of SWS secretion rate, with each box showing median, interquartile range, and upper and lower quartiles. Dots in the figure represent outliers. *P < 0.05, Mann–Whitney *U*‐test.

The overall median SWS secretion rate was 1.74 (1.24–2.38) mL min^–1^ and 4% of all participants had hyposalivation with respect to SWS (≤0.7 mL min^–1^). Women, those who took ≥4 medications, those with type II diabetes, and those who had undergone radiation therapy to the head/neck region had a significantly lower median SWS secretion rate than their counterparts (Figure 2A–E). Current smokers had a significantly lower median SWS secretion rate than former smokers but not when compared with never smokers (Figure 2B). Hyposalivation with respect to SWS showed significant association with gender, smoking status, number of medications taken, and type II diabetes (Table 3). A significantly greater proportion of current smokers (10%) than of former smokers (2%) had hyposalivation with respect to SWS, but the difference was not statistically significant compared with never smokers (5%). Furthermore, a significantly greater proportion of those who took ≥4 medications (8%) than of those who took 1–3 medications (2%) had hyposalivation with respect to SWS, but the difference was not statistically significant compared with those who took no medications (3%).

The overall median CODS was 2 (Table 3). Current smokers and those who had received radiation therapy to the head/neck region had a significantly higher median CODS than their counterparts.

### Relationship between xerostomia and hyposalivation

We found a positive, strong, and significant correlation between the responses to the standard xerostomia question (never/occasionally/frequently/always) and the SXI‐D score (*r*_s_ = 0.73). Both the standard xerostomia question and the SXI‐D score were significantly correlated with UWS and SWS secretion rates, but the correlation was strongest for UWS. The correlation between the standard xerostomia question and UWS secretion rate was negative and weak, but statistically significant (*r*_s_ = −0.20). The same was shown for the correlation between UWS secretion rate and the SXI‐D score (*r*_s_ = −0.23). Three percent of participants had both often/always dry mouth and hyposalivation with respect to UWS, while 85% of the participants had neither condition.

There was a positive, moderate, and significant correlation between UWS and SWS secretion rates (*r*_s_ = 0.48). Two percent of the participants had hyposalivation with respect to both UWS and SWS and 90% had neither condition.

The CODS showed a positive, weak, but significant, correlation with the standard xerostomia question (*r*_s_ = 0.22) and the SXI‐D score (*r*_s_ = 0.22). Furthermore, the CODS showed a negative, weak, but significant, correlation with the UWS secretion rate (*r*_s_ = −0.30) and the SWS secretion rate (*r*_s_ = −0.17).

### Regression models

The number of medications taken was significantly associated with xerostomia in both the crude and adjusted analyses, while the presence of rheumatic disease and experience with radiation therapy to the head/neck were found to be confounding factors (Table 4). Medication intake (≥4 medications) was associated with a 4.4‐fold increased risk of xerostomia compared with those of the reference categories, after adjusting for rheumatic disease and radiation therapy.

**TABLE 4:** Logistic regression model for xerostomia with number of medications as the main exposure variable.

Independent variables	Unadjusted	Adjusted
	OR (95% CI)	OR (95% CI)
Medications (no.)		
0^a^	1	1
1–3	2.4 (0.9–6.6)	2.0 (0.7–5.5)
≥4	**4.9 (1.8**–**13.6)**	**4.4 (1.6**–**12.6)**
Rheumatic disease		
No^a^	1	1
Yes	**2.3 (1.1**–**4.9)**	**2.3 (1.0**–**5.0)**
Radiation head/neck		
No^a^	1	1
Yes	**13.3 (2.9**–**61.5)**	**12.5 (2.6**–**60.6)**

^a^ Reference category; values with *P* < 0.05 are shown in bold text.

Tables 5 and 6 present the linear regression model for UWS and SWS secretion rates. The number of medications taken showed no association with UWS secretion rate in either the unadjusted or the adjusted model, while medication intake (≥4 medications) was associated with a decreased SWS section rate.

**TABLE 5:** Linear regression model for unstimulated whole saliva (UWS) secretion rate with number of medications as the main exposure variable.

Independent variables	Unadjusted	Adjusted
	β‐coefficient (95% CI)	β‐coefficient (95% CI)
Medications (no.)		
0^a^	0	0
1–3	−0.02 (−0.07 to 0.02)	−0.01 (−0.06 to 0.04)
≥4	−0.05 (−0.10 to 0.01)	−0.05 (−0.10 to 0.01)
Gender		
Male^a^	0	0
Female	**−0.10 (−0.13 to −0.06)**	**−0.10 (−0.14 to −0.06)**
Radiation head/neck		
No^a^	0	0
Yes	**−0.20 (−0.36 to −0.04)**	**−0.20 (−0.38 to −0.02)**

Note

Constant = 0.66.

As a result of the high number of unusual and influential data (outliers), failure of the data to follow a normal distribution, and heteroscedasticity of the residuals, the data were square root transformed.

^a^ Reference category; values with *P* < 0.05 are shown in bold text.

**TABLE 6:** Linear regression model for stimulated whole saliva (SWS) secretion rate with number of medications as the main exposure variable.

Independent variables	Unadjusted	Adjusted
	β‐coefficient (95% CI)	β‐coefficient (95% CI)
Medications (no.)		
0^a^	0	0
1–3	−0.01 (−0.08 to 0.06)	0.02 (−0.05 to 0.09)
≥4	**−0.13 (−0.22 to −0.05)**	**−0.09 (−0.18 to −0.01)**
Gender		
Male^a^	0	0
Female	**−0.08 (−0.14 to −0.02)**	**−0.10 (−0.15 to −0.04)**
Type II diabetes		
No^a^	0	0
Yes	**−0.22 (−0.35 to −0.08)**	**−0.17 (−0.30 to −0.05)**
Radiation head/neck		
No^a^	0	0
Yes	**−0.42 (−0.62 to −0.22)**	**−0.41 (−0.58 to −0.24)**

Note

Constant = 1.42.

As a result of the high number of unusual and influential data (outliers), failure of the data to follow a normal distribution, and heteroscedasticity of the residuals, the data were square root transformed.

^a^ Reference category; values with *P* < 0.05 are shown in bold text.

## DISCUSSION

This paper describes the occurrence of dry mouth and factors associated with this condition in a sample of 65‐yr‐old people living in Oslo. To our knowledge, the present study is one of only a few studies that focus on both subjective and objective dry mouth findings based on a comprehensive selection of measurements in a general population of young‐elderly people.

The overall prevalence of xerostomia in the present study was 10%, which is lower than found in previous studies in the same age group. JOHANSEN and coworkers performed a study on 65‐yr‐old Swedish people and found a prevalence of 15% for xerostomia (29). A study from Australia, using the same question for xerostomia as in the current study, showed that 20% of the participants within the age group 65–69 yr had ‘frequently’ or ‘always’ dry mouth (10). Furthermore, in 2009, EKBACK and coworkers reported the prevalence of xerostomia to be 30% in 65‐yr‐old Norwegians from the western part of Norway (30). However, as the definition of xerostomia used in the Norwegian study was slightly different from that used in the present study, the prevalence of xerostomia between studies is not directly comparable. Studies in younger age groups have shown a prevalence of xerostomia similar to that reported in the present study for 65‐yr‐old people; a study from Finland showed a prevalence of xerostomia of 11% among a group of 55‐yr‐old adults (20), and a study from New Zealand showed a prevalence of xerostomia of 10% among a group of 32‐yr‐old adults (25). These findings may suggest that age alone does not have a strong, direct effect on xerostomia. In addition, the use of different questionnaires to map xerostomia can also influence the results and makes it more challenging to compare the findings from different studies.

Previous studies have used several different definitions for hyposalivation (10, 18, 20, 31). In the present study, we chose the definition according to the 2002 classification criteria for Sjögren's syndrome (UWS secretion rate of ≤0.1 mL min^–1^) (26). The prevalence of hyposalivation with respect to UWS was 8% in our study. This is lower than reported in previous studies carried out on the young‐elderly/elderly age groups. ANTTILA and coworkers used the same definition for hyposalivation as in the current study and reported a prevalence of hyposalivation of 16% among 55‐yr‐old Finns (20). Studies using a slightly lower threshold for hyposalivation (<0.1 mL/min^–1^) showed a prevalence of hyposalivation of 12%–47% in different age groups ranging from 65–86 yr (9, 10, 31). In this context, if a threshold of hyposalivation of <0.1 mL/min^–1^ had been used in the current study, the prevalence of hyposalivation would have been reduced to 5%.

The prevalence of hyposalivation with respect to SWS was 4% in the present study. KONGSTAD and coworkers found a prevalence of hyposalivation among Danes, 65–74 yr of age, of 4% in men and 5% in women, which was similar to that reported in the present study (18). However, as in the study by KONGSTAD *et al*., the threshold for hyposalivation (SWS ≤0.5 mL min^–1^) was lower than that used in the present study, it can be speculated that the prevalence of hyposalivation would have been higher if they had used the same threshold as that in the present study. Many studies on older age groups have shown a higher prevalence of SWS (11%–31%) than reported in the present study (5, 8, 11, 31).

Medication use and general health of study participants are factors that can be causally related to dry mouth (8, 20, 21), and will vary between study populations. This, in addition to different definitions of xerostomia and hyposalivation, may partly explain the different prevalence estimates of these two conditions reported in the studies discussed above. Furthermore, the fact that new and improved drugs have fewer side effects may also contribute to the differences in the prevalence of dry mouth observed between studies from different periods in time.

Previous literature has highlighted the need for an increased focus on the relationship between xerostomia and hyposalivation (6). In the present study, only 3% of the participants had both ‘hyposalivation with respect to UWS’ and ‘xerostomia’, which is equivalent to one in five of those who had either condition. This proportion was somewhat larger than in the study by THOMSON and coworkers (10), in which one in six participants were reported to have both conditions. The combination of ‘hyposalivation with respect to SWS’ and ‘xerostomia’ occurred in only 0.7% of the current study population and the two conditions were not significantly associated, the latter being in accordance with the report by SREEBNY & VALDINI (32). The low correlation reported between xerostomia and hyposalivation suggests that the aetiology of xerostomia is complex, and that certain qualities of saliva, such as viscosity and the ability to lubricate mucosal surfaces, may play important roles. Limitations of the present study in this respect were that the composition and viscosity of saliva were not investigated.

Xerostomia and hyposalivation are common manifestations of some rheumatic diseases that affect the salivary glands, such as Sjögren's syndrome (33). In the current study, the presence of rheumatic disease was significantly associated with xerostomia but not with hyposalivation (UWS and SWS). In these subjects, both the rheumatic disease and the medical treatment may have affected the composition, but not the secretion rate of saliva, inducing xerostomia as a result of changes in the quality of the saliva (34). However, information on the type of rheumatic disease was not collected in the present study, thus limiting analysis of the relationship between rheumatic diseases and dry mouth.

Radiation therapy to the head/neck region is associated with a high risk of damage to the salivary glands (35). In the current study, individuals who had undergone radiation therapy had significantly lower median UWS and SWS secretion rates than those who had not had any radiation therapy. Both xerostomia and hyposalivation were more prevalent among those who had undergone radiation therapy to the head/neck region, although this relationship was not significant for hyposalivation. This could partly be a result of the low number of subjects included in this study.

The effect of medications on dry mouth is a complex phenomenon. Certain medications have dry mouth as a direct side effect, but interactions and additive effects may occur when using combinations of several different medications. Furthermore, it can be challenging to distinguish between side effects of medications on dry mouth and those of the underlying medical conditions (6). Twenty‐five percent of the participants took four or more medications, and this was significantly associated with having both xerostomia and hyposalivation. The logistic regression analysis confirmed that taking four or more medications compared with taking no medications was significantly associated with xerostomia. In addition, the linear regression analysis showed that those taking four or more medications had a lower SWS secretion rate than those who took no medications. These findings support the fact that taking four or more medications can have a direct, negative effect on xerostomia and stimulated saliva secretion rate. The prevalence of dry mouth in this study was quite low considering that 26% of the study participants took four or more medications. However, the type of medication and duration of use were not assessed in the present study.

Compared with the other participants, a greater proportion of those with type II diabetes had hyposalivation with respect to UWS and SWS. In accordance with the study by CHAVEZ and coworkers, they had a significantly lower median stimulated salivary secretion rate, but this association was not found for unstimulated saliva (36). As discussed by CHAVEZ and coworkers, these findings can be explained by the fact that diabetes can lead to autonomic neuropathies and microvascular changes that reduce the ability to respond to a salivary stimulus; therefore, the stimulated, but not the unstimulated, salivary secretion rate is affected. Furthermore, a previous study showed that individuals with type II diabetes more commonly experience xerostomia than their counterparts (37). However, in the present study and in the study by CHAVEZ *et al*., xerostomia was not prevalent in individuals with type II diabetes. More detailed data on the duration of type II diabetes and the level of blood glucose control were not collected in the present study. Such data may have provided a basis for more specific analyses of the effect of type II diabetes on salivary conditions.

The female participants in the present study had lower median UWS and SWS secretion rates than the male participants. This may be explained by the fact that women, in general, have smaller saliva glands than men (38), in addition to postmenopausal hormonal changes that can affect the glands (39). However, hyposalivation (SWS) was significantly more common among men than women in the present study, which is in contrast to previous findings (8, 11, 18). This may be related to the fact that 71% of those with type II diabetes in our study population were men. Taking four or more medications and having undergone radiation therapy to the head/neck were also more common among the male participants. In the present study, xerostomia was not associated with gender, although many studies have found that xerostomia is more common in women (8, 10, 18, 19, 20, 21).

The present study has some potential limitations. First, the response rate was 58%, meaning that there was a sizable proportion of non‐responders. As a result of restrictions from the Ethics Committee, we were not permitted to ask potential study participants why they declined to participate. A second potential limitation was selection bias. Therefore, to explore potential selection bias, the gender distribution and education level of the study population were compared with the corresponding proportions of the target population (based on register data from Statistics Norway). The gender distribution was similar, but the proportion with higher education in the current study population was higher than the average in the target population. This may have affected the prevalence estimates; however, the level of education did not show a significant association with either subjective or objective measures of dry mouth in this study.

Third, a self‐administered questionnaire was used to assess smoking habits, presence of diseases, use of medications, and symptoms of dry mouth. Therefore, these data are dependent on the responder's interpretation of the questions and their ability to recall or identify the requested information, potentially resulting in recall bias.

Finally, some factors may have affected the saliva samples. All participants were asked if they had fasted for the hour before the appointment, and 4% replied that they had not. This could have influenced the measured salivary secretion rates. Despite this, saliva was collected from all participants and it was found that the median and IQR values for the UWS secretion rate among those who did not fast (0.39 [0.16–0.48] mL min^–1^) were only slightly different from those who did fast (0.34 [0.20–0.54] mL min^–1^). However, some of the UWS secretion values among those who had not fasted were only slightly above 0.1 mL min^–1^, and these study participants may have been classified incorrectly as not having hyposalivation. Furthermore, considering the fact that the measurements were performed between 8 am and 3 pm, diurnal variations in salivary secretion rate may also have affected the results. Regarding salivary collection time, GILL and coworkers found no significant differences in salivary secretion rates between collection times (40). However, their study was performed in a younger study population (mean age ± SD: 24 ± 4 yr), and they tested only the unstimulated salivary secretion rate over a time period of 1–6 min, during which saliva was collected.

In conclusion, hyposalivation and xerostomia were infrequent among the 65‐yr‐old study population from Oslo, Norway. However, clinicians should be especially aware of the saliva status in patients taking four or more medications, those with type II diabetes, and those who have undergone radiation therapy to the head/neck region. Considering the low correlation between xerostomia and hyposalivation, not only the quantity of saliva, but also its quality, should be investigated in future studies examining xerostomia and hyposalivation.

## CONFLICT OF INTEREST

There are no conflicts of interest.

## AUTHOR CONTRIBUTION

Conception and design: My Tien Diep, Janicke Liaaen Jensen, Rasa Skudutyte‐Rysstad, Alix Young, Anne Thea Tveit Sødal, Lene Hystad Hove; Data collection: My Tien Diep, Rasa Skudutyte‐Rysstad, Anne Thea Tveit Sødal, Lene Hystad Hove; Analysis and interpretation of data: My Tien Diep, Janicke Liaaen Jensen, Rasa Skudutyte‐Rysstad, Alix Young, Anne Thea Tveit Sødal, Beáta Éva Petrovski, Lene Hystad Hove; Drafting and revising of the manuscript: My Tien Diep, Janicke Liaaen Jensen, Rasa Skudutyte‐Rysstad, Alix Young, Anne Thea Tveit Sødal, Beáta Éva Petrovski, Lene Hystad Hove.
